# Novel pathogenic variant in a mild case of type B molybdenum cofactor deficiency: case report and literature review

**DOI:** 10.1186/s12920-024-02027-x

**Published:** 2024-12-18

**Authors:** Morgan Kinsinger, Jelena Ivanisevic, Divakar S. Mithal

**Affiliations:** 1 Northwestern University Feinberg School of Medicine, Chicago, IL 60611 USA; 2 Ann and Robert H. Lurie Children’s Hospital of Chicago, Chicago, IL 60611 USA

**Keywords:** Molybdenum cofactor deficiency (MoCD), MoCD type B, MOCS2, Molybdenum cofactor (MoCo), Molybdopterin (MPT) synthase, Globus pallidus (GP) injury, Exome sequencing, Case report

## Abstract

**Background:**

Molybdenum cofactor deficiency (MoCD) is a rare metabolic disorder caused by pathogenic variants in the highly conserved biosynthetic pathway of molybdenum cofactor (MoCo), resulting in sulfite intoxication. MoCD may present in a clinically severe, fatal form marked by intractable seizures after birth, hyperekplexia, microcephaly and cerebral atrophy, or a later onset form with a more varied clinical course. Three types of MoCD have been described based on the effected gene along the MoCo synthesis pathway: type A (*MOCS1)*; type B (*MOCS2* or *MOCS3)* and type C (*GPHN*). The *MOCS2* gene is bicistronic, encoding the small (MOCS2A) and large (MOCS2B) subunits with an overlapping coding region. This case report describes a patient with the first known variant causative of mild disease in the overlapping bicistronic region (c.263 G > C) and the first ever described in the highly conserved C-terminal glycine-glycine motif of MOCS2A.

**Case presentation:**

The patient developed normally until age 12 months when she presented in the setting of acute illness with developmental regression, low serum uric acid, and MRI with bilateral globus pallidus (GP) injury. Exome sequencing identified a homozygous variant of unknown significance in the *MOCS2* gene and the diagnosis of MoCD type B was confirmed by the patient’s low serum uric acid coupled with elevated urine sulfocysteine and associated metabolites, resulting in gene reclassification. Nearly four years after her initial presentation she has demonstrated progress in language and motor domains, consistent with a mild phenotype of MoCD.

**Conclusions:**

The case emphasizes challenges in identifying atypical forms of rare diseases, the importance of exome sequencing to identify mild cases of MoCD, and the ongoing challenges with understanding the *MOCS2* gene. While one FDA approved treatment exists for MoCD type A, further research into the mechanisms of phenotype-genotype differences among this patient population may aid in additional therapeutic options for MoCD.

**Supplementary Information:**

The online version contains supplementary material available at 10.1186/s12920-024-02027-x.

## Background

Molybdenum cofactor deficiency (MoCD) is a rare, autosomal recessive metabolic disorder first described in 1978 [1]. MoCD is caused by pathogenic variants in genes of the highly conserved biosynthetic pathway of molybdenum cofactor (MoCo) from GTP [2]. In humans, MoCo is essential for its redox function in four key enzymes: sulfite oxidase, aldehyde oxidase, xanthine oxidoreductase, and mitochondrial amidoxime-reducing component (mARC). MoCo deficiency, therefore, leads to a toxic accumulation of the metabolites upstream of these enzymes including sulfite, taurine, S-sulfocysteine and thiosulfate. Of these, sulfite accumulation is the most clinically deleterious, leading to progressive neurological damage potentially through disrupted DNA alkylation although the mechanisms are not yet fully understood [3, 4]. Clinical presentations of MoCD range from mild to severe and the determinants of disease severity have not been elucidated from enzyme function studies completed to date [5, 6].

Disease categorizations for MoCD are variable in the literature, but loosely fall into two categories as described by Johannes et al [7]. Patients with severe MoCD typically present with intractable seizures shortly after birth, feeding difficulties, hyperekplexia, microcephaly and cerebral atrophy, severe developmental delay, and distinctive facial features [6]. Mild cases have similarity to Leigh Syndrome, often presenting with an intercurrent illness within the first 2 years of life after a period of relatively normal development, and follow a more varied clinical course [8–10]. The incidence of MoCD is likely underestimated due to misdiagnosis of hypoxic ischemic encephalopathy (HIE) or cerebral palsy and the high early fatality rate among the severe phenotype [11–13]. MoCD diagnostic criteria include elevated urine sulfite, S-sulfocysteine, taurine, thiosulfate, xanthine and hypoxanthine, and decreased urine and serum levels of uric acid due to loss of xanthine dehydrogenase function [8]. Brain MRI differ significantly between patients but usually reveal symmetric lesions, diffusely affecting a variety of brain structures, with abnormalities ranging from poor myelination and gliotic changes to atrophy and cystic necrosis [14].

**Fig. 1:**
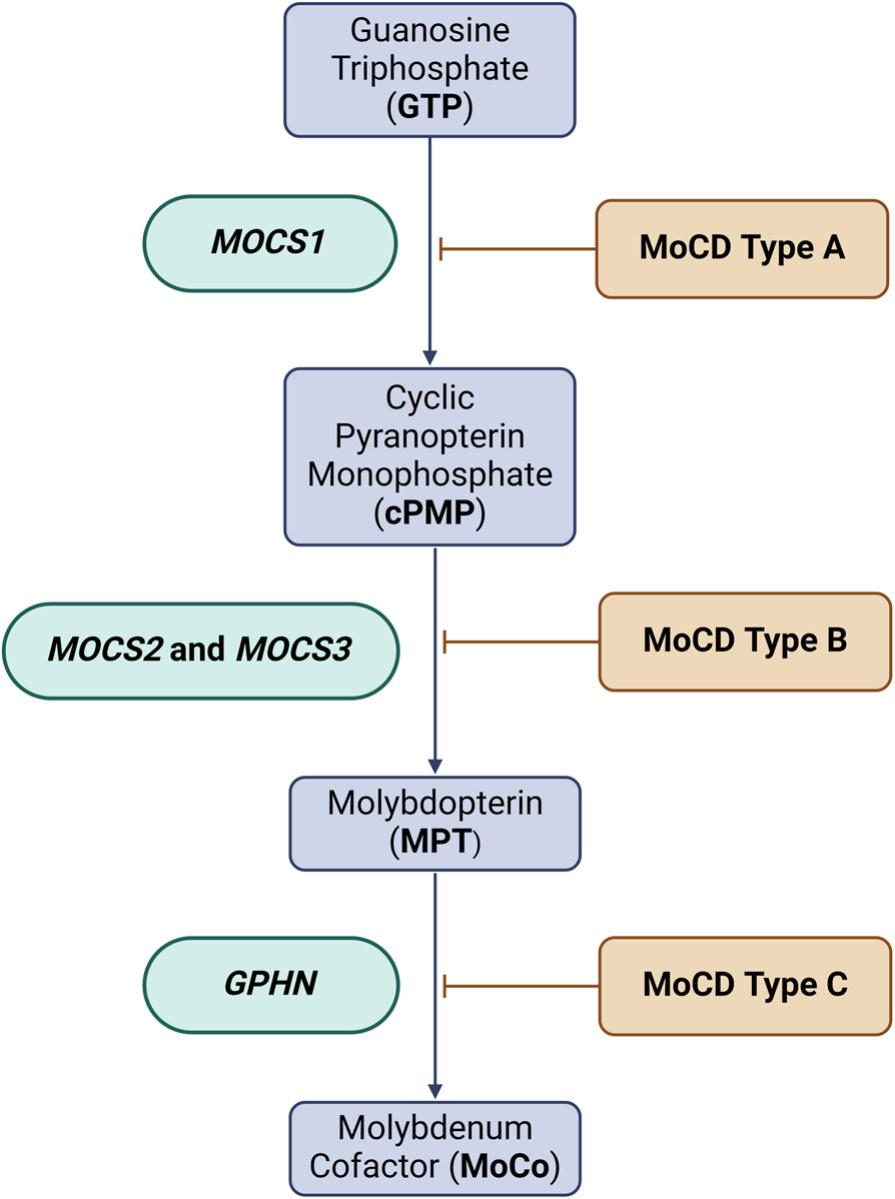
Molybdenum cofactor biosynthesis pathway

There are three distinct forms of MoCD based on the function of the deficient enzyme in the MoCo biosynthetic pathway: type A (*MOCS1)*; type B (*MOCS2* or *MOCS3)* and type C (*GPHN*) (See Fig. 1). The *MOCS2* gene consists of 7 exons on chromosome 5q and is bicistronic, with two overlapping open reading frames (ORFs) encoding the small (MOCS2A) and large (MOCS2B) subunits of molybdopterin (MPT) synthase [15]. MPT synthase is a heterotetrametric enzyme which converts cyclic pyranopterin monophosphate (cPMP) to MPT by transferring two sulfide groups, a crucial step in MoCo synthesis and the basis of MoCD type B [16]. While type A is potentially responsive to supplementation with cPMP, which is downstream of *MOCS1*, treatment must be initiated as soon as possible for any meaningful benefit. Because cPMP is unable to be converted to MPT in type B cases, these patients have not been shown to respond to cPMP supplementation as summarized in a recent review by Schwahn et. al [17–19].

We present a case of a one-year old patient with an acute but mild clinical presentation of MoCD type B due to a novel pathogenic variant, c.263 G > C in *MOCS2*. Imaging demonstrated bilateral acute injury to the globus pallidus. Diagnosis of MoCD was confirmed by low uric acid, elevated urine sulfocysteine, and exome sequencing. A literature review of all MoCD type B cases confirmed by genetic testing to date, with all available clinical and genetic data, are summarized in Supplementary Table S1 (online only).

## Case presentation

The patient presented as a 12-month old female with apparently normal development who suffered an episode of acute suppurative otitis media leading to altered mental status, nuchal rigidity, photophobia and emesis, without fever. Upon admission she was afebrile, irritable and drowsy, with low tone and negative Kernig and Brudzinski signs.

The medical history of the patient was significant for birth via cesarean section at 39 and 6/7 weeks due to difficult labor. Mother was 25 years old with gestational diabetes and pre-eclampsia. Birth weight was 7 lbs., 13 oz. Apgar scores were 9 at 1 min and 5 min. At the time of birth, she was noted to have difficulty feeding resulting in a 9.4% decrease in weight by day 3 of life. Muscle tone was within normal limits, and Moro and grasp reflexes were present. No other clinical concerns were noted until a routine office visit at 10 months noted strabismus, for which a referral was placed. At one year of age, she was noted to be cruising, feeding herself and otherwise developing normally. The patient was the second child of first-degree consanguineous Pakistani parents. The elder sister was unaffected.

**Fig. 2:**
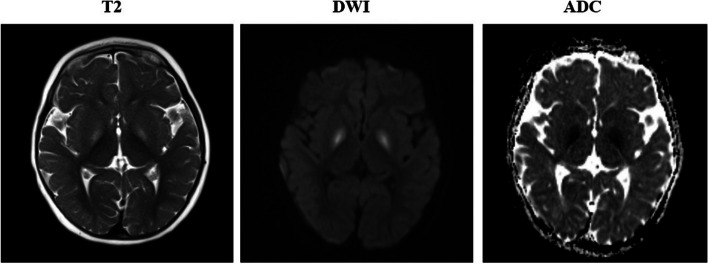
MRI at presentation age 12 months, T2 sequences demonstrating increased signal of the globus pallidus bilaterally. DWI and ADC demonstrating bilateral diffusion restriction in the globus pallidus

Upon her acute presentation at 12 months, a head CT noted areas of hypodensity within the basal ganglia. A follow up MRI demonstrated increased T2 FLAIR signal within the bilateral globus (see Fig. 2). Diffusion weighted imaging (DWI) (Fig. 2) and apparent diffusion coefficient (ADC) (Fig. 2) confirmed a pattern of restricted diffusion consistent with acute injury. MRI spectroscopy to test for lactate was not completed on initial imaging due to a high suspicion for carbon monoxide poisoning. CSF meningitis/encephalitis PCR panel, CSF and blood cultures, and SARS-CoV2 test were all negative. CSF protein was 12 mg/dL, WBC count was less than 1 cell/mm^3^, RBC count was 8 cell/mm^3^, and glucose was 62 mg/dL. Carboxyhemoglobin (2.7%) and methemoglobin (1.3%) were slightly elevated on admission, but normalized on repeat testing. Urine toxicology screen was negative for methanol, ethanol, isopropanol, and acetone. Screening for peroxisomal disorders with a very-long-chain fatty acid profile was normal. Urine organic acids noted elevated 3-hydroxybutyric and acetoacetic acids, consistent with ketosis, but not diagnostic of a recognized inborn error of organic acid metabolism. Plasma acylcarnitine assay revealed elevated Tetradecanoylcarnitine C14 and Octadecanoylcarnitine C18, which were of unclear significance but possibly related to fasting. Serum amino acids were unrevealing, with a normal alanine of 378 umol/L and citrulline of 21 umol/L, while L-cystine was decreased at 7umol/L. Serum pyruvic acid was 0.12 mmol/L and serum lactate was 1.0 mEq/L, which were both normal. Although the patient never experienced events concerning for seizures, an EEG obtained for screening purposes showed 6 Hz PDR bursts of slowing without epileptiform activity, consistent with mild global neurologic dysfunction. The patient was discharged after 4 days showing significant improvement but remained non-ambulatory with persistent fussiness and diffuse hypotonia, with a contingent diagnosis of carbon monoxide toxicity.

**Fig. 3:**
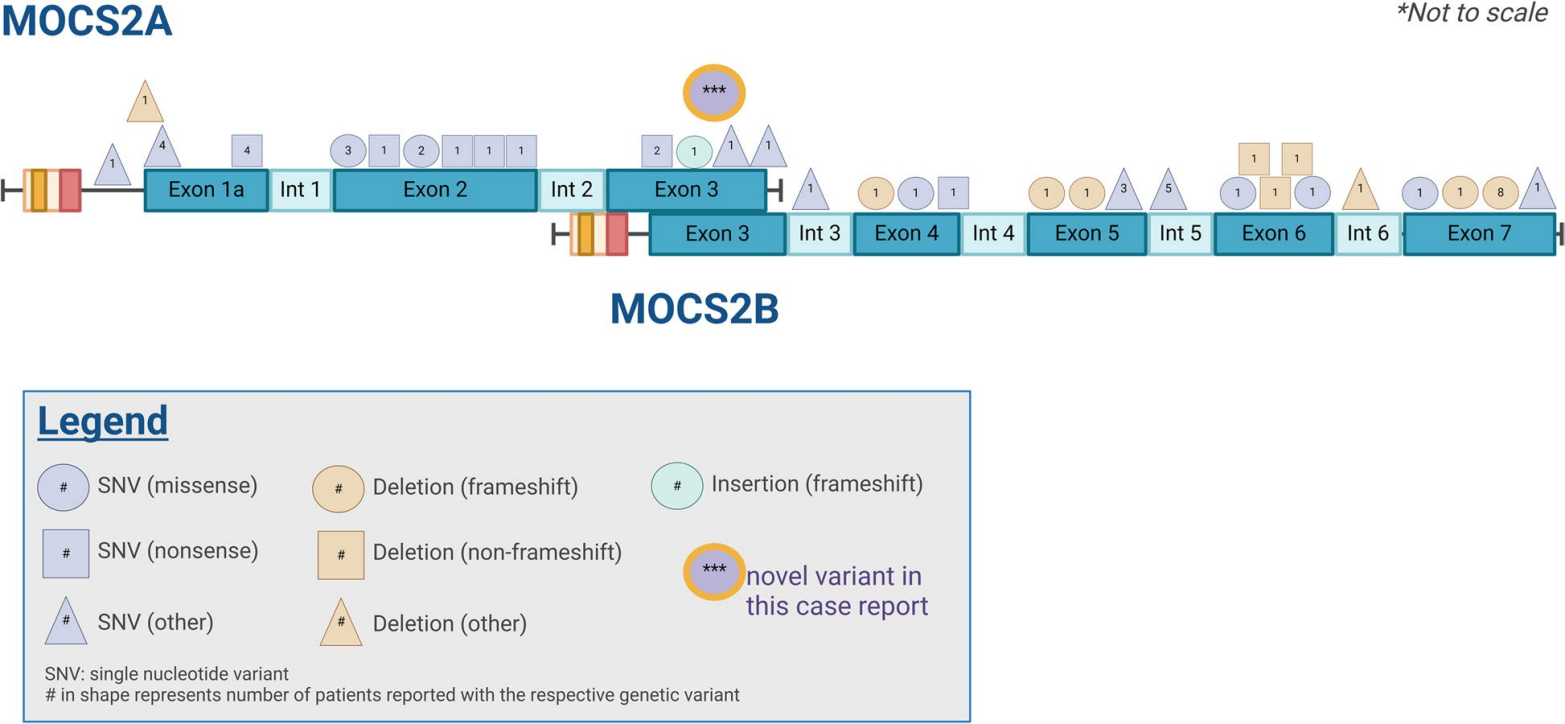
Diagram of MOCS2 gene with all known pathogenic variants

On outpatient follow up, the patient remained unable to walk and had not regained her pre-hospitalization baseline. Outpatient notes from the pediatrician describe orange deposits in the patient’s diapers. Additional metabolic and genetic testing was recommended. Quantitative urine organic acid evaluation showed elevated taurine to 5495.1 pmol/g creatine (normal 168–2446), and a slightly elevated 2-methylglutaconic acid/3-methylglutaconic acid ratio. The latter was suggestive of ECHS1 deficiency, but additional urine testing including a C4-acylcarnitine of 0.47 mmol/mol creatinine (reference value < 3.0 mmol/mol creatinine) and C5-DC acylcarnitine of 0.48 mmol/mol creatinine (reference value < 1.54 mmol/mol creatinine) were normal. Exome sequencing identified a homozygous variant of unknown significance in the *MOCS2* gene (c.263 G > C, MOCS2A:p.G88A, MOCS2B:p.D26H) which was a candidate gene for the patient’s clinical phenotype. There were no other mitochondrial or nuclear variants of significance identified on exome or mitochondrial sequencing. The identified missense variant is located at coding strand position 263 which lies in the overlapping region of the two bicistronic subunits MOCS2A and MOCS2B (Fig. 3). The variant is predicted to lead to amino acid change Gly > Ala at protein position 88 in MOCS2A, and Arg > His in MOCS2B. The diagnosis of MoCD type B was confirmed by our patient’s elevated urine sulfocysteine (121.5 mmol/mol creatinine) and low uric acid (< 0.2 mg/dL). Gene variant classification, however, required additional testing for urine S-sulfocysteine (elevated at 60 mmol/ mol creatinine, normal < 5), hypoxanthine (increased at 81 mmol/ mol creatinine, normal < 30) and xanthine (increased at 290 mmol/ mol creatinine, normal < 21), which gave final confirmation of the diagnosis and resulted in reclassification of the gene to “Likely Pathogenic.”

Initially after hospitalization, the patient demonstrated persistent regression of walking skills, with spasticity, fisting, and continued irritability. In the subsequent 2 years she demonstrated a slow but persistent course of recovery. Nearly two years after her initial presentation she is able to speak multiple words together, roll over, and ambulate with minimal assistance. She exhibits intermittent, alternating exotropia and mild hyperopic astigmatism not requiring correction. She received therapies through early intervention with a plan to transition to her school district. The family of the patient described in this case report provided written informed consent for this publication.

## Discussion and conclusions

Thirty three pathogenic variants have so far been identified across all 7 exons and 3 introns of *MOCS2* (Fig. 3). Efforts have been made to distinguish which known MoCD variants correlate with the mild phenotype, based on the hypothesis that these variants exhibit residual enzymatic function [5, 20]. However, to date research has been limited by small sample sizes of patients with this ultra-rare disease and additional enzyme assays are needed to determine disease severity. Among the five patients with pathogenic variants described in the overlapping region of exon 3, our patient is this first who presented with a mild phenotype. This patient’s variant is the first ever discovered in the highly conserved C-terminal glycine-glycine motif of MOCS2A. PolyPhen-2 predicts that our patient’s variant is “probably damaging” in MOCS2A and “benign” in MOCS2B [21]. The MOCS2A C-terminus has been shown to form the active site of MPT synthase which acts as a sulfur donor [16]. As these residues have been shown to be essential to MOCS1A function, our findings suggest they may be similarly necessary in MOCS2A [22].

**Fig. 4:**
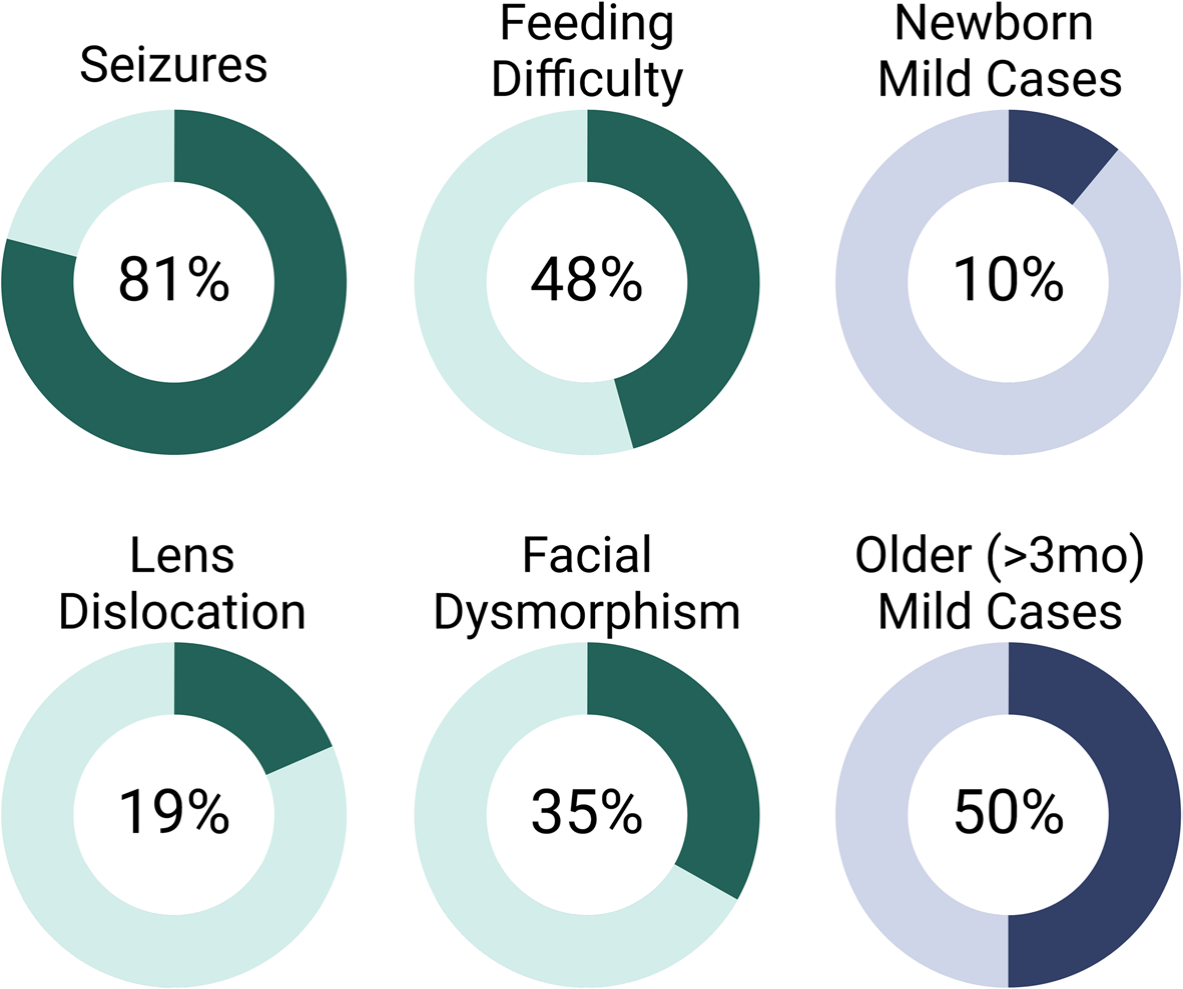
Summary of key clinical phenotypes of MoCD Type B caused by MOCS2 variants

Of the 49 cases confirmed by genetic testing to date, only 8 cases (16%) of mild MoCD type B have been identified including the patient described in this report (see Supplementary Table S1, online only). Only 31 of the 49 previously reported cases included data regarding clinical phenotypes. The majority (81%) of cases reported seizure activity, while only 19% noted lens dislocation. 35% described facial dysmorphism. 48% of cases reported feeding difficulty at birth, a finding which has not generally been included in general clinical descriptions of the disease (Fig. 4). Four cases including the patient presented here were noted to have yellow or orange xanthine stones in their urine which may represent a novel diagnostic aid [23–25].

Among the 52 known cases of type B MoCD, our patient is rare in that she had normal health and development until an intercurrent illness at age 12 months when she presented acutely with isolated GP injury. The MRI findings were non-diagnostic and led to a presumptive diagnosis of carbon monoxide toxicity. The initial metabolic testing including lactate and pyruvate, plasma amino acids, urine organic acids, peroxisomal fatty acid profile and acylcarnitine profile, were nondiagnostic. In the outpatient setting, despite the lack of initial metabolic findings, exome sequencing was pursued which identified the candidate variant in the *MOCS2* gene. Ultimately, the diagnosis of MoCD was confirmed by testing for urine S-Sulfocysteine and associated metabolites, and at the time of publication the VUS classification is being reconsidered. We found that patients who presented after 3 months of age were more likely to demonstrate a mild phenotype (50%), as compared with those presenting as newborns (10%) (see Fig. 4). Misko et al., provide an alternative way to view MoCD by grouping them with sulfite oxidase deficiency to create a spectrum of neurologic disease secondary to sulfite toxicity [5]. The current patient would fall into the milder phenotype Class II subgroup. As the authors of that manuscript suggest, the current patient serves to raise awareness and promote diagnosis of these ultra-rare diseases.

Variants implicated in mild cases were evenly distributed among the 7 exons and were all point variations except for 1 frameshift deletion in a heterozygous patient, indicating that specific genetic loci are not predictors of mild disease. However, among the 5 variants in the overlapping bicistronic region of exon 3, the novel variant reported here is the only mild case and the only case which presented after 3 months of age. Given that the variant is also housed within the highly conserved C-terminal glycine-glycine motif of MOCS2A, it suggests a gap in our current understanding of the factors contributing to pathogenicity of *MOCS2* variants.

Limitations to this study included the lack of reported clinical phenotype data for 18 of the 49 cases described in the literature (see Supplementary Table S1). The limited number of cases prevented adequately powered analysis of statistically significant differences between early- and late-presenting groups.

In addition to identifying a novel pathogenic variant in the bicistronic region of *MOCS2*, the present case expands on the milder phenotype MoCD Type B. The case suggests that milder cases of MoCD could be underdiagnosed due to nonspecific clinical or radiographic findings, and there is likely benefit to rapid deployment of broad genetic testing for patients with undifferentiated metabolic abnormalities, particularly in the ICU setting. Further research is needed to characterize the mild MoCD or Class II phenotypes, investigate the mechanisms that account for the clinical differences among patients, and identify future directions for treatment and prognostication.

## Supplementary Information

Supplementary Material 1.

## Data Availability

The genetic  variant analyzed during the current study is available in ClinVar repository with the primary accession code VCV003233444.1.

## Abbreviations

ADC: Apparent diffusion coefficient
cPMP: Cyclic pyranopterin monophosphate
DWI: Diffusion weighted imaging
GP: Globus pallidus
HIE: Hypoxic ischemic encephalopathy
mARC: Mitochondrial amidoxime-reducing component
MoCD: Molybdenum cofactor deficiency
MoCo: Molybdenum cofactor
MPT: Molybdopterin
ORF: Open reading frame
