# Supplementary material for: Novel pathogenic variant in a mild case of type B molybdenum cofactor deficiency: case report and literature review
Source: BMC Med Genomics. 2024 Dec 18;17:292. doi: 10.1186/s12920-024-02027-x (PMC11658218; doi:10.1186/s12920-024-02027-x)
Supplement: Supplementary file 1 — Supplementary Material 1. [file 12920_2024_2027_MOESM1_ESM.zip › Supplementary/Supplementary Table S1 Citations 6.2.24.docx]

**Supplementary Table S1 Citations:**

1. Hughes, Fairbanks, L., Simmonds, H. A., & Robinson, R. O. (1998). Molybdenum cofactor deficiency – phenotypic variability in a family with a late‐onset variant. *Developmental Medicine and Child Neurology, 40*(1), 57–61. *doi*:10.1111/j.1469-8749.1998.tb15357.
2. Alkufri, Harrower, T., Rahman, Y., Hughes, E., Mundy, H., Knibb, J. A., Moriarty, J., Connor, S., & Samuel, M. (2013). Molybdenum cofactor deficiency presenting with a parkinsonism-dystonia syndrome*. Movement Disorders, 28*(3), 399–401. *doi*:10.1002/mds.25276
3. Reiss, Dorche, C., Stallmeyer, B., Mendel, R. R., Cohen, N., & Zabot, M. T. (1999). Human Molybdopterin Synthase Gene: Genomic Structure and Mutations in Molybdenum Cofactor Deficiency Type B. *American Journal of Human Genetics, 64*(3), 706–711. *doi:*10.1086/302296
4. Boles, Ment, L. R., Meyn, M. S., Horwich, A. L., Kratz, L. E., & Rinaldo, P. (1993). Short-term response to dietary therapy in molybdenum cofactor deficiency*. Annals of Neurology, 34*(5), 742–744. *doi:*10.1002/ana.410340520
5. Johnson, Coyne, K. E., Rajagopalan, K. V., Van Hove, J. L. K., Mackay, M., Pitt, J., & Boneh, A. (2001). Molybdopterin synthase mutations in a mild case of molybdenum cofactor deficiency. *American Journal of Medical Genetics, 104*(2), 169–173. *doi:*10.1002/1096-8628(20011122)104:23.0.CO;2-8
6. Reiss, & Johnson, J. L. (2003). Mutations in the molybdenum cofactor biosynthetic genes MOCS1, MOCS2, and GEPH. *Human Mutation, 21*(6), 569–576. *doi:*10.1002/humu.10223
7. Leimkuhler, Charcosset, M., Latour, P., Dorche, C., Kleppe, S., Scaglia, F., Szymczak, Schupp, P., Hahnewald, R., & Reiss, J. (2005). Ten novel mutations in the molybdenum cofactor genes MOCS1 and MOCS2 and in vitro characterization of a MOCS2 mutation that abolishes the binding ability of molybdopterin synthase. *Human Genetics, 117*(6), 565–570. *doi:*10.1007/s00439-005-1341-9
8. Hahnewald, Leimkühler, S., Vilaseca, A., Acquaviva-Bourdain, C., Lenz, U., & Reiss, J. (2006). A novel MOCS2 mutation reveals coordinated expression of the small and large subunit of molybdopterin synthase. *Molecular Genetics and Metabolism, 89*(3), 210–213. *doi:*10.1016/j.ymgme.2006.04.008
9. Per, Gümüş, H., Ichida, K., Çağlayan, O., & Kumandaş, S. (2007). Molybdenum cofactor deficiency: Clinical features in a Turkish patient. *Brain & Development (Tokyo. 1979), 29*(6), 365–368. *doi:*10.1016/j.braindev.2006.10.007
10. Sie, De Jonge, R. C. ., Blom, H. J., Mulder, M. F., Reiss, J., Vermeulen, R. J., & Peeters-Scholte, C. M. P. C. . (2010). Chronological changes of the amplitude-integrated EEG in a neonate with molybdenum cofactor deficiency. *Journal of Inherited Metabolic Disease, 33*(SUPPL. 3), 401–407. *doi*:10.1007/s10545-010-9198-z
11. Reiss, & Hahnewald, R. (2011). Molybdenum cofactor deficiency: Mutations in GPHN, MOCS1, and MOCS2. *Human Mutation, 32*(1), 10–18. *doi:*10.1002/humu.21390
12. Struys, Nota, B., Bakkali, A., Al Shahwan, S., Salomons, G. S., & Tabarki, B. (2012). Pyridoxine-dependent epilepsy with elevated urinary -amino adipic semialdehyde in molybdenum cofactor deficiency.(Case Reports*). Pediatrics (Evanston), 130*(6), E1716–. *doi*:10.1542/peds.2012-1094
13. Kikuchi, Hamano, S., Mochizuki, H., Ichida, K., & Ida, H. (2012). Molybdenum Cofactor Deficiency Mimics Cerebral Palsy: Differentiating Factors for Diagnosis. *Pediatric Neurology, 47*(2), 147–149. *doi:*10.1016/j.pediatrneurol.2012.04.013
14. Stence, Coughlin, C. R., Fenton, L. Z., & Thomas, J. A. (2013). Distinctive pattern of restricted diffusion in a neonate with molybdenum cofactor deficiency*. Pediatric Radiology, 43*(7), 882–885. *doi*:10.1007/s00247-012-2579-8
15. Edwards, Roeper, J., Allgood, C., Chin, R., Santamaria, J., Wong, F., Schwarz, G., & Whitehall, J. (2015). Investigation of molybdenum cofactor deficiency due to MOCS2 deficiency in a newborn baby*. Meta Gene, 3*, 43–49. *doi*:10.1016/j.mgene.2014.12.003
16. Megahed, Nicouleau, M., Barcia, G., Medina-Cano, D., Siquier-Pernet, K., Bole-Feysot, C., Parisot, M., Masson, C., Nitschke, P., Rio, M., Bahi-Buisson, N., Desguerre, I., Munnich, A., Boddaert, N., Colleaux, L., & Cantagrel, V. (2016). Utility of whole exome sequencing for the early diagnosis of pediatric-onset cerebellar atrophy associated with developmental delay in an inbred population. *Orphanet Journal of Rare Diseases, 11*(1), 57–57. *doi:*10.1186/s13023-016-0436-9
17. Zaki, Selim, L., EL-Bassyouni, H. T., Isaa, M. Y., Mahmoud, I., Ismail, S., Girgis, M., Sadek, A. A., Gleeson, J. G., & Abdel Hamid, M. S. (2016). Molybdenum Cofactor and Isolated Sulphite Oxidase Deficiencies: Clinical and Molecular Spectrum Among Egyptian Patients. *European Journal of Paediatric Neurology, 20*(5), 714–722. *doi:*10.1016/j.ejpn.2016.05.011
18. Durousset, C., Gay, C., Magnin, S., Acquaviva, C., & Patural, H. (2016). Encéphalopathie néonatale grave liée à un défaut d’activité de la sulfite-oxydase par fficia en cofacteur molybdène [Sulfite oxidase activity deficiency caused by cofactor molybdenum deficiency: A case of early severe encephalopathy]. *Archives de pediatrie : organe fficial de la Societe francaise de pediatrie, 23*(3), 292–296. *doi*:10.1016/j.arcped.2015.12.005
19. Yoganathan, Sudhakar, S., Thomas, M., Kumar Dutta, A., Danda, S., & Chandran, M. (2018). Novel Imaging Finding and Novel Mutation in an Infant with Molybdenum Cofactor Deficiency, a Mimicker of Hypoxic-Ischaemic Encephalopathy. *Iranian Journal of Child Neurology, 12*(2), 107–112. *doi*:10.22037/ijcn.v12i2.12671
20. Duman F., & Gençpinar P.. (2018). Molibden Kofaktör Eksikliği ve Lens Dislokasyonu: Olgu Sunumu*. J Glaucoma Cataract, 13*, 38-41.
21. Arican, Gencpinar, P., Kirbiyik, O., Bozkaya Yilmaz, S., Ersen, A., Oztekin, O., & Olgac Dundar, N. (2019). The Clinical and Molecular Characteristics of Molybdenum Cofactor Deficiency Due to MOCS2 Mutations. *Pediatric Neurology, 99*, 55–59. *doi:*10.1016/j.pediatrneurol.2019.04.021
22. Scelsa, Gasperini, S., Righini, A., Iascone, M., Brazzoduro, V. G., & Veggiotti, P. (2019). Mild phenotype in Molybdenum cofactor deficiency: A new patient and review of the literature. *Molecular Genetics & Genomic Medicine, 7*(6), e657–n/a. *doi:*10.1002/mgg3.657
23. Hannah-Shmouni, MacNeil, L., Potter, M., Jobling, R., Yoon, G., Laughlin, S., Blaser, S., & Inbar-Feigenberg, M. (2019). Severe cystic degeneration and intractable seizures in a newborn with molybdenum cofactor deficiency type B. *Molecular Genetics and Metabolism Reports, 18*, 11–13. *doi*:10.1016/j.ymgmr.2018.12.003
24. Karunakar, Krishnamurthy, S., Kasinathan, A., Hariharan, R., & Chidambaram, A. C. (2020). Renal stones in an infant with microcephaly and spastic quadriparesis: Answers. *Pediatric Nephrology (Berlin, West), 35*(6), 987–989. *doi:*10.1007/s00467-019-04449-7
25. Jezela-Stanek, Blaz, W., Gora, A., Bochenska, M., Kusmierska, K., & Sykut-Cegielska, J. (2020). Proteins Structure Models in the Evaluation of Novel Variant (C.472_477del) in the MOCS2 Gene. *Diagnostics (Basel), 10*(10), 821–. *doi*:10.3390/diagnostics10100821
26. Lin, Liu, Y., Chen, S., Zhu, J., Huang, Y., Lin, Z., & Chen, S. (2021). A neonate with molybdenum cofactor deficiency type B*. Translational Pediatrics, 10*(4), 1039–1044.  *doi*:10.21037/tp-20-357
27. Lee, H. F., Hsu, C. C., Chi, C. S., & Tsai, C. R. (2022). Genotype-Phenotype Dissociation in Two Taiwanese Children with Molybdenum Cofactor Deficiency Caused by MOCS2 Mutation. *Neuropediatrics, 53*(3), 200–203. *doi:*10.1055/s-0041-1736181
28. Tian, X. J., Li, X., Fang, F., Liu, Z. M., Wu, W. J., Liu, K., & Sun, S. Z. (2021). *Chinese journal of pediatrics, 59*(2), 119–124. *doi:*10.3760/cma.j.cn112140-20200911-00866
29. Lee, Dandamudi, R., Granadillo, J. L., Grange, D. K., & Kakajiwala, A. (2021). Rare cause of xanthinuria: a pediatric case of molybdenum cofactor deficiency B. *CEN Case Reports, 10*(3), 378–382. *doi:*10.1007/s13730-021-00572-3
30. Yan, Huang, L., Sun, L., & Ding, X. (2022). Ocular characteristics of a 6-year-Old boy with molybdenum cofactor deficiency type B. *American Journal of Ophthalmology Case Reports, 27*, 101586–. *doi:*10.1016/j.ajoc.2022.101586
31. Pedro Maneira Sousa, Teresa Campos, Luísa Sampaio, & Ana Vilan. (2022). Multicystic Brain and Refractory Neonatal Seizures*. Portuguese Journal of Pediatrics (Online), 53*(1). *doi:*10.25754/pjp.2022.21901
